# Supplementary material for: Antenna Cleaning Is Essential for Precise Behavioral Response to Alarm Pheromone and Nestmate–Non-Nestmate Discrimination in Japanese Carpenter Ants (Camponotus japonicus)
Source: Insects. 2021 Aug 28;12(9):773. doi: 10.3390/insects12090773 (PMC8471180; doi:10.3390/insects12090773)
Supplement: Supplementary file 1 [file insects-12-00773-s001.zip › insects-1224879-supplementary.pdf]

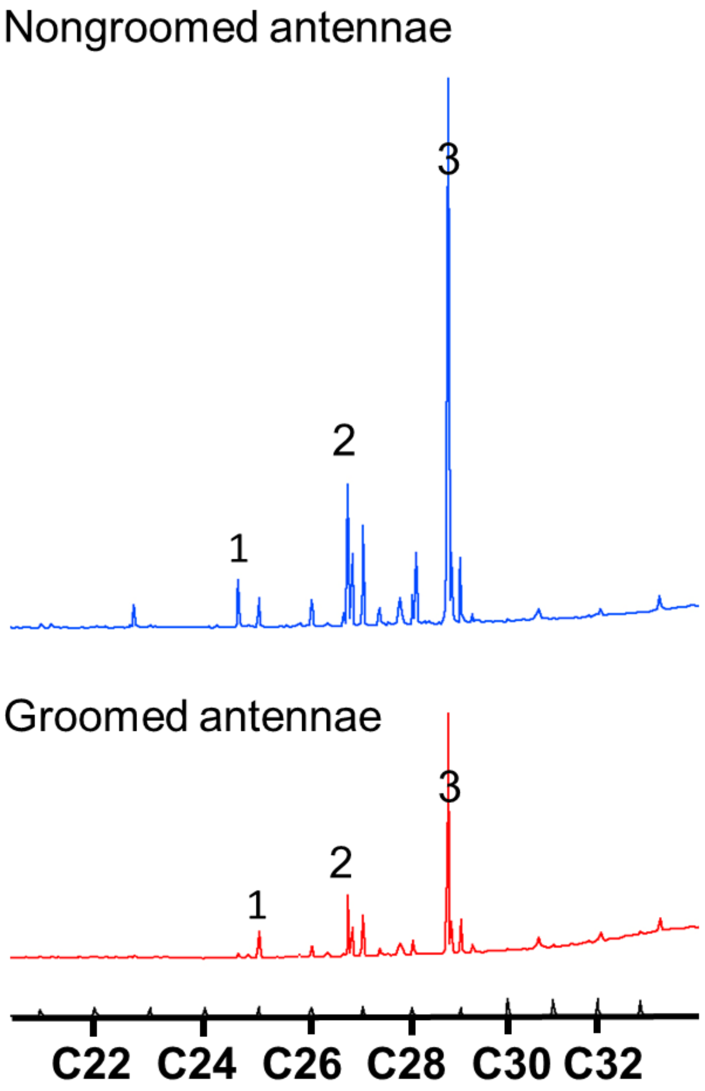

Figure S1. Gas-chromatograms of nongroomed and groomed antennal cuticular hydrocarbons of *C. japonicus*. Cuticular hydrocarbons (CHCs) accumulate on nongroomed antennae of *C. japonicus* workers. Isolated antennae from 10 workers that were self-grooming limited for 24 h or nonlimited were dipped in *n*-hexane for 5 min to extract antennal CHCs. Extracted CHCs were purified by a silica gel column and applied to the GC analysis. Detail method and peaks are as reported by Ozaki et al. (2005) [33]: *n*-pentacosane (Peak 1), 9-heptacosene (Peak 2), and 9-nonacosene (Peak 3), for example.
